# Addressing Rotator Cuff-Related Shoulder Pain: Findings from a Greek Regional Observational Study Utilizing a Clinical Case Scenario

**DOI:** 10.3390/clinpract15020030

**Published:** 2025-01-31

**Authors:** Eleftherios Paraskevopoulos, Anna Christakou, Andrew Smythe, Eleni Kapreli, Maria Papandreou, Charalambos Papacharalambous, Kyriakos Pavlou, George M. Pamboris

**Affiliations:** 1 Biomechanics Laboratory, Department of Physiotherapy, University of Peloponnese, 23100 Sparta, Greece; achristakou@uniwa.gr; 2 Physiotherapy Department, School of Primary and Allied Health Care, Faculty of Medicine, Nursing and Health Science, Monash University, Frankston, VIC 3199, Australia; andy.smythe@monash.edu; 3 Clinical Exercise Physiology and Rehabilitation Research Laboratory, Physiotherapy Department, University of Thessaly, 35132 Lamia, Greece; ekapreli@uth.gr; 4 Laboratory of Advanced Physiotherapy, Department of Physiotherapy, University of West Attica, 12243 Athens, Greece; mpapand@uniwa.gr; 5 Department of Health Sciences, School of Sciences, European University Cyprus, 2404 Nicosia, Cyprus; c.papacharalambous@external.euc.ac.cy (C.P.); k.pavlou@external.euc.ac.cy (K.P.); g.pamboris@euc.ac.cy (G.M.P.)

**Keywords:** Greece, physiotherapy, rotator cuff, shoulder pain, survey

## Abstract

**Background**: Rotator cuff-related shoulder pain (RCRSP) is a prevalent musculoskeletal issue, encompassing various shoulder conditions. While exercise typically forms the foundation of conservative treatment, there exists ongoing discourse regarding the effectiveness and role of passive treatments. International guidelines recommend initial conservative management, with surgery considered only after failed conservative treatment. However, recent studies reveal discrepancies between recommended practices and actual clinical management. The aim of the study was to assess current practices in managing RCRSP among Greek physiotherapists, with a focus on understanding the alignment of these practices with international guidelines for conservative treatment. **Methods**: A cross-sectional survey was conducted among Greek physiotherapists to assess current practices in managing RCRSP. The survey, adapted from previous studies, collected demographic data and assessed clinical reasoning through a vignette-based approach. Responses were analyzed for alignment with guideline-recommended care. **Results**: Out of over 9000 contacted physiotherapists, 163 responded. A majority expressed a specific interest in shoulder pain (85%). Patient education (100%) and exercise (100%) were widely endorsed, with limited support for imaging (44%), injection (40%), and surgery (26%). Younger respondents were less inclined towards surgical referral (*p* = 0.001). Additionally, adjunctive interventions like mobilization (66%) and massage therapy (58%) were commonly employed alongside exercise and education. Treatment duration typically ranged from 6 to 8 weeks, with exercises reviewed weekly. **Conclusions**: The study highlights a consistent preference for conservative management among Greek physiotherapists, aligning with international guidelines. However, there are variations in practice, particularly regarding adjunctive interventions and exercise prescription parameters. Notably, there is a disparity between recommended and actual use of certain modalities.

## 1. Introduction

Shoulder discomfort ranks among the most prevalent musculoskeletal issues, affecting approximately 15% to 30% of people at any given moment [1,2]. Additionally, rotator cuff-related shoulder pain (RCRSP) is observed in approximately 70% of patients experiencing shoulder discomfort [1,2]. RCRSP is a broad term encompassing various shoulder conditions, such as subacromial pain (impingement) syndrome, rotator cuff tendinopathy, and symptomatic partial and full-thickness rotator cuff tears [3]. There has been contention that RCRSP is a more suitable designation than traditional diagnoses rooted in pathoanatomic and structural pathologies [4]. This is because pinpointing a specific structure as the primary source of a patient’s shoulder pain remains highly challenging [5].

Conservative treatment for RCRSP typically includes a regimen of strengthening exercises, stretching, and mobility exercises targeting the shoulder, thoracic, and cervical spine [1,6]. Exercise is considered the cornerstone of managing shoulder issues, supported by numerous systematic reviews emphasizing its statistical and clinical effectiveness in reducing pain and improving function [1,6]. Nonetheless, prior research trials have demonstrated favorable outcomes by incorporating manual therapy (MT) alongside exercise interventions for RCRSP patients [7]. It has been suggested that MT may elicit hypoalgesic effects or promote the restoration of normal biomechanics by enhancing the shoulder’s range of motion [1,7]. However, a recent systematic review reported no notable distinction in outcomes between solely exercising and combining exercise with manual therapy during short and long-term follow-ups regarding both shoulder pain and function [1].

A synthesis of various international guidelines for RCRSP suggests initial management strategies such as activity modification (adjusting activities that may exacerbate the condition) and education, along with clinician-guided exercises spanning 6–12 weeks [8,9,10]. In cases where there is no improvement following the initial management phase, imaging may be considered [8,9,10]. Similar guidance is provided regarding the timing and indication for injection interventions, with most guidelines recommending injections after 6–12 weeks only if initial management proves ineffective [8,10,11].

Research indicates comparable outcomes between surgical intervention and clinician-led exercise, yet surgery entails significantly greater risks and costs than exercise treatment [8,9,10,12,13,14,15]. Consequently, guidelines suggest that surgical consultation should be considered only after a 12-week trial of initial management has been unsuccessful [8,9,10]. Moreover, as recently reported [11], previous studies examining physiotherapy practices for RCRSP in the United Kingdom, Belgium, the Netherlands, France, and Australia have demonstrated that education and exercise are commonly implemented, aligning with guideline recommendations [2,11,16,17]. However, these studies also reveal the presence of non-recommended treatments such as TENS and ultrasound in clinical practice, as well as considerable variability in specific exercise parameters (such as dosage, frequency, and acceptable levels of symptom provocation during and after exercise) and diverse reasoning behind the selection of exercise parameters [2,11,16,17]. Recent research on patient-reported management for RCRSP has identified high rates of early imaging, injections, and surgery, which contradict recommended practices [2].

To date, no studies have investigated whether physiotherapists in Greece adhere to international guidelines for the management of RCRSP. Therefore, the main objective of this study was to examine current physiotherapy practices for RCRSP among physiotherapists in Greece and to compare these practices with guideline-recommended care.

## 2. Materials and Methods

### 2.1. Study Design

This study is nota cross-sectional survey that was adapted from previous published studies after permission from the authors [2,11]. The study protocol was approved by the ethics committee of the University of Peloponnese (approval number: 6242/20-03-2024, approval date: 20 March 2024). The survey was conducted online between April 2024 and May 2024. It was promoted via various social media platforms, including Facebook, Instagram, and LinkedIn, targeting Greek physiotherapy groups.

Moreover, the Panhellenic Physiotherapists’ Association informed all members via Viber and email about the survey, which allowed us to reach more respondents through their contact list. Respondents who did not identify as physiotherapists working in Greece were excluded from the analysis. Only fully completed responses were considered for analysis. It is worth noting that all responses were collected anonymously, and no personal data was gathered during the survey process.

### 2.2. Survey Questionnaire

The survey was initially developed by Bury and Littlewood [16] and later modified by Smythe, Rathi, Pavlova, Littlewood, Connell, Haines, and Malliaras [2]. The later version of the survey was translated into Greek and slightly adjusted to suit the Greek context. It was created using Microsoft Forms and was subject to cognitive interviewing with a sample of two Greek physiotherapists to assess clarity and identify potential online operational issues. Minor adjustments were made based on feedback from the cognitive interviewing. To increase completion rates, we changed some open-ended questions to multiple-choice ones. The potential answers for the multiple choice questions were taken from the original work of Smythe, Rathi, Pavlova, Littlewood, Connell, Haines, and Malliaras [2], and Riera, Smythe and Malliaras [11], who performed content analysis to analyse data from the open-ended questions.

**Figure 1:**
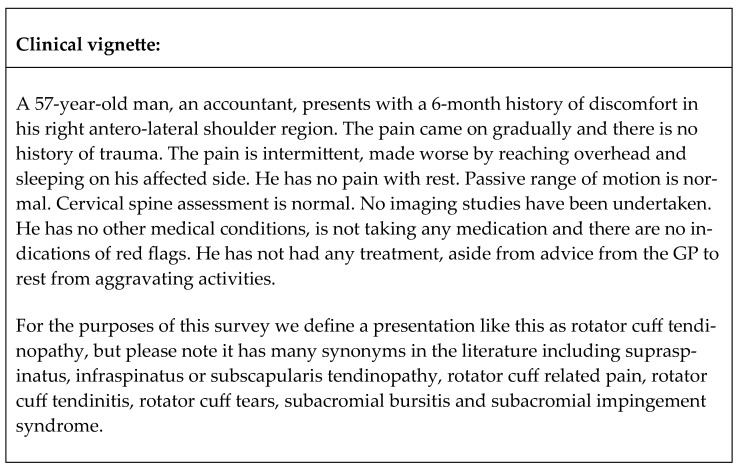
Clinical vignette.

The survey comprised 32 questions divided into three sections. The first section included demographic information, including age, gender, level of clinical experience, work setting (e.g., private practice), location (e.g., rural), the highest level of qualification, and whether participants had a special interest in shoulder pain (questions 1–10). The second section investigated clinical reasoning in RCRSP through a clinical vignette depicting a common presentation of RCRSP, adapted from the work of Bury and Littlewood [16] (Figure 1). Clinical vignettes are recognized as valid tools for reflecting on clinical practice and decision-making [18]. Subsequent multiple-choice and open-ended questions explored treatment decision-making, as well as the frequency and duration of treatment related to the vignette (questions 11–23). The third and last section included additional multiple-choice questions (questions 24–32) to investigate specific practices of Greek physiotherapists regarding exercise prescription and education.

### 2.3. Assessment of Optimal Treatment Approaches

In the determination of recommended care, responses given by participants were assessed against pertinent guidelines to ascertain whether they aligned with current recommended management practices [8,10,17,19]. A comprehensive overview of evidence from these guidelines and reviews, addressing queries arising from the vignette, is presented in previous research studies [2,11].

### 2.4. Statistical Analysis

All data collected through the survey platform, Microsoft Forms, were transferred to Excel 2016 (Microsoft Corp., Redmond, WA, USA) for analysis. The survey encompassed demographic variables such as experience, post-graduate education, work setting, work location, and special interest in shoulder pain. Specifically focusing on the clinical vignette, the survey reported the frequency of referrals for imaging, injections, and surgical opinions, as well as the implementation of education, exercise, and adjunct interventions for the entire participant cohort. Furthermore, the association between years of experience, work location, work setting, special interest, and referral decisions was examined using statistical tests such as the Chi-square test or Fisher’s exact test (when Chi-square test assumptions were not met) in IBM Statistical Package for the Social Sciences (SPSS) 23.00, with data exported from Excel 2016 (Microsoft Corp.). The significance level for all analyses was set at 0.05.

Open-ended responses were transcribed verbatim and organized using Excel 2016 (Microsoft Corp.) for qualitative data management. A qualitative content analysis methodology was employed, allowing for the condensation of large datasets into manageable themes [20]. Two researchers systematically identified units of meaning by thoroughly examining each response and generating initial codes manually. These codes were initially organized into preliminary categories based on the focus of the open-ended questions and common topics in physiotherapy management. Subsequently, the codes were refined into distinct categories, and a descriptive column was added to the Excel spreadsheet, following established practices [21]. Additionally, the frequency of categorical descriptions was analyzed to provide further insight.

## 3. Results

The questionnaire was sent to > 9000 registered physiotherapists via email (through the member’s list of the Panhellenic Association of Physiotherapists) or through social media. Data from 163 respondents were gathered and processed further for statistical analysis. Table 1 presents the demographic characteristics of the cohort. The majority of participants identified as female (82 out of 163; 50%), expressed a specific interest in shoulder pain (139 out of 163; 85%), worked in Central Greece (91 out of 163; 56%), practiced in private settings (123 out of 163; 75%), had received post-graduate training in the form of seminar (78 out of 163; 48%), and had been qualified for ≤5 years (56 out of 163; 34%). Moreover, most of the respondents had been working with shoulder patients for ≤5 years (64 out of 163; 39%) and had been treating an average of 6–10 patients with shoulder complaints per month (69 out of 163; 42%). Regarding the respondents’ clinical interests, the majority were primarily treating patients with musculoskeletal and other complaints (109 out of 163; 67%).

### 3.1. Care Recommendations for the Clinical Vignette

The majority of participants, 91 out of 163 (56%), did not advocate for imaging. Those with post-graduate education were less inclined to suggest imaging referrals (Chi-square = 11.67, *p* = 0.009), particularly those with seminar education. Imaging referrals were not found to be associated with work location, work experience (overall or in shoulder rehabilitation), or age.

The majority of respondents, accounting for 97 out of 163 (60%), did not recommend referral for injection. Conversely, 35% (57 out of 163) were either unsure or would advocate for injection (6.0%, 9 out of 163). Factors such as work setting, work experience (overall or in shoulder rehabilitation), or age were not found to be significantly correlated with the decision-making process regarding injection referral.

Most respondents, accounting for 120 out of 163 (74%), did not recommend referral for surgery. Conversely, 20% (33 out of 163) were unsure or would advocate for injection (6.0%, 10 out of 163). Factors such as work setting or work experience (overall or in shoulder rehabilitation) were not found to be significantly correlated with the decision-making process regarding surgical referral. However, younger individuals, particularly those between the ages of 25 and 34, were found to be less inclined to recommend surgical management (Chi-square = 29.42, *p* = 0.001).

### 3.2. Physiotherapy Management

All of the respondents expressed a preference for educating patients on recommended physiotherapy management. Activity modification was commonly recommended, with 135 out of 163 respondents (82%) endorsing it. Additionally, 125 out of 163 respondents (76%) emphasized educating patients on risk factors, while 119 and 118 out of 163 respondents (73 and 72%) highlighted the importance of suggesting proper treatment with physiotherapeutic modalities and explaining the relationship between pathology and pain. In contrast, education on the timing and role of imaging was less common, with only 45 out of 163 respondents (27%) suggesting it. Similarly, recommendations for injections and surgical intervention were infrequent, with only 14 out of 163 respondents (8%) and 20 out of 163 respondents (12%), respectively (Table 2).

All respondents (163 out of 163; 100%) recommended exercise, aligning with guideline-recommended care. The most frequently recommended types of exercises were those targeting the rotator cuff (131 out of 163; 80%), scapula exercises (106 out of 163; 65%), exercises for the kinetic chain (117 out of 163; 71%), isometric exercises (116 out of 163; 71%), and proprioception exercises (87 out of 163; 53%). Aerobic exercise (14%), stretching (39%), and isokinetic exercise (17%) were the least frequent responses (Table 2).

Written or printed instructions were the most commonly used methods of education delivery, employed by 36% of respondents (105 out of 163). Verbal instructions (89 out of 163; 30%), video recordings (66 out of 163; 40%), and links to online resources (32 out of 163; 19%) were reported as less common in clinical practice (Table 2).

In terms of adjunctive management, mobilization was the preferred approach for most respondents, with 109 out of 163 (66%) indicating its use. Less commonly employed strategies included massage therapy (95 out of 163; 58%) and electrotherapy (92 out of 163; 56%). The least recommended strategies were rest (49 out of 163; 30%), manipulations (23 out of 163; 14%), and suggestions for paracetamol or anti-inflammatory medication use (40 out of 163; 24%) (Table 2).

Regarding treatment duration, the majority of respondents (40%, 66 out of 163) indicated that they would expect to treat a patient with RCRSP for up to 6 weeks. A smaller percentage, 26% (43 out of 163), anticipated seeing patients for 8 weeks. Only 3% of respondents (6 out of 163) indicated that they would expect a treatment duration of 12 months (Table 2).

In terms of the frequency of reviewing patients within this timeframe, 55% (90 out of 163) reported that they would check or modify the exercises of an RCRSP patient on a weekly basis. Less common frequencies included reviews every two weeks (35%, 58 out of 163) or every three weeks (6%, 11 out of 163). Only one respondent stated they would not modify exercises after the initial prescription (Table 2).

### 3.3. Provision of Instructions Regarding Exercise

In terms of exercise guidance, the majority of respondents believed that experiencing pain during exercise was acceptable, but it should not escalate by more than 2–3 points on the Visual Analog Scale (VAS) (101 out of 163; 62%). Regarding load management during exercise, most respondents indicated that weight should be determined by their symptoms and should not result in pain exceeding 4–5 points on the VAS (72 out of 163; 44%). Furthermore, the majority of respondents suggested that sets and repetitions should be chosen based on patients’ symptoms and irritability (122 out of 163; 75%). Similarly, respondents expressed that exercise frequency should be determined by patients’ symptoms and irritability (56 out of 163; 34%). Lastly, most respondents recommended adjusting exercise intensity by either increasing or decreasing the weight (69 out of 163; 43%) (Table 3).

### 3.4. Open-Ended Questions Analysis

Responses to open-ended questions were analyzed through content analysis, focusing on clinical indications for imaging, injections, or surgical intervention. Concerning imaging, 156 out of 163 respondents (95%) indicated that imaging was unnecessary given the clinical scenario. One respondent noted, “*As long as there has been no prior pharmacotherapy or conservative rehabilitation, clinical imaging may be considered later if symptoms persist despite conservative care.*” This suggests that the respondent believed imaging was not currently necessary for the patient. The remaining respondents did not provide responses.

Regarding injections, 147 out of 163 respondents (90%) stated that injections were not necessary based on the clinical scenario. Only a minority [5 out of 163; 3%] believed injections, such as cortisone or Platelet-Rich Plasma (PRP), might benefit the patient. For instance, one respondent suggested, “*In cases of persistent pain, I would recommend PRP and cortisone in rare instances.*” The remainder did not respond to this question.

Regarding surgery, 142 out of 163 respondents (87%) stated that it was unnecessary based on the clinical scenario. Only a minority [8 out of 163; 5%] believed that indicators such as the inability to raise the arm, intense pain, or a tendon tear warranted surgery. For instance, one respondent mentioned, “*Inability to lift a limb and intense, unbearable continuous pain experienced by the patient*”, while another respondent stated, “*In case of a total rupture of a tendon, which affects the movement of the shoulder.*” The remaining respondents did not respond to this question.

## 4. Discussion

The study provides valuable insights into the current practices of Greek physiotherapists in managing RCRSP and compares them with international guidelines. Most respondents favoured conservative management, which was consistent with established guidelines emphasizing exercise as the cornerstone of treatment. However, there were notable variations in practice, particularly regarding referrals for imaging and injections, adjunctive interventions, exercise prescription parameters, and the recommended use of certain modalities.

### 4.1. Referral for Imaging, Injection, and Surgery

Aligned with international guidelines [9,10], the study revealed that the majority of Greek physiotherapists did not advocate for imaging, injections, or surgery, favoring conservative approaches in the absence of red flags. This aligns with the principle of initiating conservative management before considering invasive interventions, reflecting a patient-centred approach focused on minimizing risks and maximizing outcomes. However, a significant proportion of physiotherapists suggested otherwise.

A large proportion (44%) recommended imaging despite the absence of red flags. Individuals with post-graduate education, particularly those who had attended seminars, were significantly less likely to recommend imaging. This finding has been previously uncovered, as newly certified practitioners are most likely to inform their practice through seminar education [22]. Moreover, more and more countries are introducing mandatory continuing education. Thus, there is a growing demand for professional development. The easiest way to receive post-graduate education is through seminars [23]. This finding is very important and highlights the need for accreditation of these seminars by university institutions and other independent evaluators with no financial interest (i.e., journal editors) [22].

In comparison with other countries, the rate of referral for imaging by Greek physiotherapists is significantly higher than that in Australia (6.4%), the United Kingdom (9%), France (12.6%), and the Netherlands (31%) [11]. Despite recent findings from Alaiti, et al. [24] which showed that shoulder pain is not related to diagnostic imaging findings, a relatively high percentage of Greek physiotherapists still recommended imaging. This is a significant finding since premature diagnostic imaging may have negative repercussions [25].

Diagnostic labeling, which involves assigning specific tissue-related diagnoses based on imaging or other diagnostic processes, can lead to over diagnosis and overtreatment of findings that may or may not be clinically relevant [26]. These labels can also affect patients’ perceptions of the severity of their condition and the necessary treatment. For instance, individuals experiencing shoulder pain related to rotator cuff issues might mistakenly believe that surgery is necessary if tendon tears are detected [27]. This phenomenon, where the interpretation of medical information negatively influences the patient, is referred to as the nocebo effect [25]. Health professionals can mitigate these negative effects on patient behaviour by appropriately contextualizing diagnostic imaging findings, which may involve challenging misconceptions [28].

Additionally, a significant percentage (41%) expressed uncertainty or advised injection despite the guidelines [9,10]. This discovery underscores the necessity for continuous professional development, as studies have revealed lasting adverse effects of corticosteroid injection (CSI), including persistent pain, degradation of tendon structure, and heightened risk of injury recurrence [29]. Moreover, the long-term efficacy of corticosteroid injection has been questioned in previous research studies [30,31].

The indicated outcomes could potentially be clarified by the distinction in Greece whereby both diagnostic imaging and injections are considered medical procedures strictly performed or prescribed by medical professionals. This contrasts with countries such as the United Kingdom or the USA, where physiotherapists began expanding their scope of practice in 1995, following formal training, to include tasks like administering intra-articular injections or ordering diagnostic imaging [32]. However, a lack of knowledge regarding guidelines may affect multidisciplinary collaboration and hinder potential future expansions of the scope of physiotherapy in Greece.

### 4.2. Patient Education

The recommended approach for managing RCRSP emphasizes the significance of patient education [9,10]. However, existing guidelines and literature often lack clear directives on the content of this education [11]. This lack of specificity is mirrored in the varied educational approaches adopted by practitioners. Furthermore, only a minority of respondents—approximately 23% for risk factor education and 17% for activity modification—did not acknowledge the importance of including these aspects in their management strategies. Notably, these findings align with similar observations in countries like Belgium/the Netherlands (20%) and Australia (15%). The significance of these results lies in the understanding that persisting with activities that exacerbate symptoms can compromise the efficacy of physiotherapy interventions and potentially contribute to the development of chronic symptoms [10,11].

Education and activity modification were commonly recommended, highlighting the importance of empowering patients with knowledge and modifying activities to manage symptoms effectively. Nevertheless, there was noticeable diversity in the choice of educational strategies employed, indicating a lack of consistency. This underscores the necessity for more precise guidelines to address this variability and ensure uniformity in educational approaches.

### 4.3. Exercise Recommendations

In line with recommended care, all respondents suggested exercise as a component of RCRSP treatment. However, variations existed in exercise types, dosage, and frequency, suggesting a need for standardization and clearer guidance in exercise prescription. Based on the findings, most respondents would direct their treatment towards the rotator cuff (80%), the kinetic chain and/or using isometrics (71%), and the scapula (65%). These findings are in line with the other countries, including Australia [2], France [11], the United Kingdom [16] and Belgium/the Netherlands [17].

The challenge of managing individuals with RCRSP is acknowledged by clinicians [6], a sentiment echoed not only in this study but also in similar studies conducted in other countries. This complexity is further underscored by recent research indicating a lack of sustained significant differences between specific motor control exercises and general resistance exercises for RCRSP [33], as well as between various combinations of education, motor control exercises, and resistance exercises [34]. Additionally, while most studies have focused on strength training and motor control exercises for RCRSP management, it appears that other factors, such as reducing kinesiophobia [35] and restoring biochemical balance in shoulder tissues, may also play pivotal roles in effective management [36].

Regarding pain management during exercise, respondents consistently recommended allowing mild discomfort ranging from 2 to 3 on the VAS scale. This aligns with research indicating potential benefits of moderate pain levels, up to 5 out of 10, compared to pain-free exercise for RCRSP management [37]. However, it is worth noting that painful exercises may be counterproductive for patients with pain-catastrophizing [38]. Although this aspect was not explicitly addressed in the clinical vignette of this study, it is inferred that respondents appropriately tailored their treatment recommendations based on pain levels. Furthermore, the suitability of exercise prescription in terms of loading, sets, and repetitions was demonstrated in the results, with most respondents suggesting individualized adjustments based on patients’ symptoms and irritability, as previously proposed [6].

### 4.4. Adjunctive Treatment Modalities

Additional treatment methods were frequently advised in the survey; however, there was significant variability in the selection of specific modalities. Manual therapy techniques, including mobilization and massage, were the most commonly cited. Specifically, 66% of the respondents recommended mobilization in response to the provided clinical vignette (File S1). This aligns with guidelines which advocate for the use of manual therapy alongside active treatment methods [8,9,10]. However, it should be mentioned that more recent research has questioned the clinical significance of adding manual therapy techniques in the management of RCRSP [1,39]. Thus, future guidelines should examine this consideration further.

Although manual therapy techniques are recommended in published guidelines, the results of this study indicate that Greek physiotherapists still heavily rely on low-value passive treatments such as electrotherapy and thermo/cryo-therapy. Despite numerous studies demonstrating the limited effectiveness of these interventions, 56% and 43% of the respondents in our study suggested the use of electrotherapy and thermo/cryo-therapy, respectively. This contrasts with findings from other countries, such as Australia [2], France [11], the United Kingdom [16] and Belgium/the Netherlands [17], where the utilization of such modalities was much lower, selected by less than 18% of the respondents. For instance, in the United Kingdom, where the scope of practice for physiotherapists has been extended, only 3% of the respondents believed that electrotherapy could effectively manage RCRSP.

This difference in practice patterns between Greek and British physiotherapists may be attributed to the predominant setting in which they work. In Greece, physiotherapists primarily operate within the private sector, while British physiotherapists are largely employed by the British National Health Service (NHS) [40]. Consequently, the use of electrotherapy or other patient-preferred therapies may still be challenging to exclude in Greek private practice settings. However, qualitative research studies are warranted to explore this aspect further. Such studies could provide insights that would be valuable for policymakers and physiotherapists in private practice, aiding in the transition towards evidence-based management of RCRSP. Moreover, the fact that physiotherapists in Greece mainly work in the private sector explains why only a small number of respondents reported working elsewhere.

### 4.5. Limitations

The study exhibits several limitations that warrant consideration. Firstly, its reliance on convenience sampling through social media platforms and professional associations may introduce sampling bias, as it potentially excludes physiotherapists who are less active or not affiliated with these groups. As a result, the findings may not fully represent the broader population of physiotherapists in Greece. Additionally, the self-reported nature of the data exposes it to response bias, where participants may provide answers influenced by social desirability or personal beliefs, potentially skewing the results. Furthermore, the generalizability of the study is constrained by its specific sample, which primarily included individuals active on social media or affiliated with professional associations, thereby not fully capturing the diversity of physiotherapy practitioners across the country. Likewise, the small overall sample, as well as the lack of expertise (36% of the sample) and experience (48% of the sample with less than 10 years of experience) in a significant number of the respondents, may be also seen as a limitation.

Another notable limitation is the lack of validation for the survey instrument. While adapted from previous studies and subject to pilot testing, the manuscript does not detail the validation process, raising concerns about the reliability and accuracy of the collected data. Moreover, the cross-sectional design employed in the study restricts the ability to establish causal relationships or observe longitudinal trends in physiotherapy practices. Longitudinal studies would offer more robust insights into changes over time and causal links between variables.

The language and cultural adaptation of the survey instrument present additional limitations. Although it was translated into Greek and adjusted for the local context, the adequacy of the translation process and the effectiveness of the cultural adaptation remain uncertain. Poor translation or cultural mismatch could undermine the validity of the findings. Moreover, self-selection bias may affect the study’s outcomes, as respondents who opt to participate may differ systematically from those who decline. This bias could impact the representativeness of the sample and the generalizability of the results.

Lastly, the scope of analysis in the manuscript may be limited, potentially overlooking other influential factors in physiotherapy practice related to RCRSP management. A more comprehensive examination of various variables and factors could provide a more nuanced understanding of clinical decision-making and treatment outcomes in this context. Addressing these limitations in future research endeavours would improve the validity, reliability, and applicability of findings related to physiotherapy practices in Greece.

## 5. Conclusions

The findings of this study underscore the consistent preference for conservative management among Greek physiotherapists in treating RCRSP, aligning with international guidelines advocating for exercise-based interventions as first-line treatment. However, variations exist in practice patterns, particularly concerning referrals for imaging, injections, and adjunctive interventions. Moving forward, efforts to standardize exercise protocols, enhance education on evidence-based practice, and promote interdisciplinary collaboration may help bridge the gap between guideline recommendations and clinical practice.

## Figures and Tables

**Table 1:** Respondents’ clinical settings, work location, and kinds of patient.

	Range	Number	Percentage
Age			
	18–24	10	6%
	25–34	63	39%
	35–44	41	25%
	45–54	35	21%
	55–64	13	8%
	65–74	1	1%
Gender			
	Male	79	48%
	Female	82	50%
	Prefer not to answer	2	1%
Years Qualified as Physiotherapist			
	0–5	56	34%
	6–10	23	14%
	11–15	24	15%
	16–20	18	11%
	21–25	16	10%
	26–30	13	8%
	>31	11	7%
Number of Patients Treated Per Month			
	<5	59	36%
	6–10	69	42%
	11–20	29	18%
	21–30	5	3%
	>30	1	1%
Work Setting			
	Private Practice	123	75%
	Public Health Facility (e.g., health centre)	9	5.5%
	Elderly Care Unit	6	3.6%
	Hospital	13	8%
	Educational Institution	8	5%
	Other	17	10.4%
Area of expertise			
	Musculoskeletal and other	109	67%
	Musculoskeletal	50	31%
	Non-musculoskeletal	2	1%
	Not working as a clinician	2	1%
Post-graduate education			
	Seminar	78	48%
	Master of Science	57	35%
	None	21	13%
	PhD	5	3%
	Other	2	1%

**Table 2:** Respondents’ answers on patient education, type of recommended exercise, instructions provided for home exercises, adjunctive management strategies, treatment duration, and frequency of reviewing the exercise programme.

	Range	Number	Percentage
Educational advice for patients			
	Information on the pathology of rotator cuff pain, including the tissues that may be involved	118	72%
	The relationship between the occurrence of tendinopathy of the rotator cuff and the occurrence of pain	81	50%
	Risk factors, such as changing activities, lifting heavy weights, age, metabolism, etc.	125	77%
	Factors that may influence pain, such as stress levels and patient beliefs/expectations	108	66%
	Proposed physical therapy management (pathology management with physical therapy means)	119	73%
	Modifying activities and body positions (e.g., work, sports) if they are painful	135	83%
	Time stages of pathology progression and indications for imaging	45	28%
	Time stages of the development of the pathology and indications for injection treatment	14	9%
	Time stages of pathology development and indications for receiving surgical repair	20	12%
	Other	12	7%
What exercise program would you recommend for this patient?			
	No exercise	0	0%
	Stretching exercises	65	40%
	Shoulder isometric exercises	116	71%
	Shoulder isotonic exercises	59	36%
	Shoulder eccentric exercises	78	48%
	Shoulder isokinetic exercises	29	18%
	Exercises targeting the scapula	106	65%
	Proprioceptive exercises	87	53%
	Exercises targeting the rotator cuff	131	80%
	Exercises for the cervical and thoracic spine	83	51%
	Exercises for the motor chain of the entire upper limb	117	72%
	Aerobic exercises	24	15%
	Other	8	5%
How do you typically instruct your patients to perform the “home” exercises?			
	Written or printed information	105	64%
	Links to online videos or websites	32	20%
	Recorded videos on their cell phone or other device	66	40%
	Verbal instructions	89	55%
What other management strategies would you recommend for this patient?			
	Counseling on taking paracetamol and anti-inflammatory drugs for pain	40	25%
	Manipulations	23	14%
	Joint mobilization	109	67%
	Μassage	95	58%
	Treatment directed at the cervical/thoracic spine	53	33%
	Taping	64	39%
	Acupuncture/dry needling technique	62	38%
	Electrotherapy	92	56%
	Thermotherapy or cryotherapy	70	43%
	Rest	49	30%
	Other	20	12%
How long would you expect a patient with reported rotator cuff pain to need physical therapy?			
	Up to 3 weeks	14	9%
	Up to 6 weeks	66	40%
	Up to 8 weeks	43	26%
	Up to 3 months	20	12%
	Up to 6 months	14	9%
	Up to 12 months	6	4%
How often would you review and possibly modify the exercise program of a patient with reported rotator cuff pain?			
	I would not suggest exercise	1	1%
	Never since my original prescription	1	1%
	At least on a weekly basis	90	55%
	About every 2 weeks	58	36%
	About every 3 weeks	11	7%
	About once a month or more	2	1%

**Table 3:** Respondents’ answers on pain during exercise, load management, repetitions and sets, and exercise progression and regression.

	Range	Number	Percentage
When formulating exercise programs, what guidelines do you typically give regarding pain during exercise?			
	Not to hurt at all when performing exercises	18	11%
	Pain is allowed when performing exercises	6	4%
	Pain should not exceed 2–3 out of 10 on the VAS scale (0–10)	101	62%
	Pain should not exceed 6–7 out of 10 on the VAS scale (0–10)	5	3%
	The pain should subside after the end of the exercise	26	16%
	The pain should subside the next day (within 24 h)	5	3%
When prescribing an exercise program, what guidelines do you usually give in terms of load/resistance level?			
	Start with a light load (e.g., dumbbell) of 1–2 kg	39	24%
	Start with a load of 60–70% of 1 Repetition Maximum	7	4%
	Determine load based on symptoms (e.g., any load that results in pain no greater than 4–5 out of 10 on the VAS scale)	72	44%
	Do not determine the load based on the fatigue it causes (e.g., load that causes significant fatigue at 12 repetitions-to-failure)	10	6%
	Determine the load based on the maximum load they can lift without negatively affecting their technique when performing the exercise	21	13%
	Determine the load based on the goal they have (e.g., exercise for strength, hypertrophy, endurance)	12	7%
When prescribing an exercise program, what instructions do you usually give in terms of reps/sets?			
	Specific set and reps for everyone (e.g., 3 sets of 12 reps)	7	4%
	Depending on the patient’s symptoms and irritability	122	75%
	Depending on the goal (e.g., 3 × 45 s hold for isometric, 3 sets × 12 repetitions for isotonic)	28	17%
	Other	4	2%
When prescribing an exercise regimen, what guidelines do you usually give in terms of frequency?			
	I suggest daily execution of the exercises	44	27%
	I suggest daily execution of the exercises 3–5 times a day	19	12%
	Several times a week (3–5 times)	24	15%
	Depending on symptoms, pain, etc.	56	34%
	Depending on the goal (e.g., strengthening, hypertrophy, etc.)	11	7%
	Depending on fatigue	2	1%
	Other	5	3%
When prescribing an exercise program, what instructions do you usually give in terms of progressing or limiting the progress of the exercises?			
	I suggest they increase/decrease the load	69	42%
	I suggest they increase/decrease sets and reps	44	27%
	I suggest they increase/decrease the range they perform the exercise	32	20%
	Other	16	10%

## Data Availability

Data are available from the corresponding author upon reasonable request.

## Supplementary Materials

The following supporting information can be downloaded at: https://www.mdpi.com/article/10.3390/clinpract15020030/s1. File S1: Synthesis of best available evidence for clinical vignette (Retrieved from Smythe et al., 2021 [2]).
