# Supplementary material for: Addressing Rotator Cuff-Related Shoulder Pain: Findings from a Greek Regional Observational Study Utilizing a Clinical Case Scenario
Source: Clin Pract. 2025 Jan 31;15(2):30. doi: 10.3390/clinpract15020030 (PMC11853849; doi:10.3390/clinpract15020030)
Supplement: Supplementary file 1 [file clinpract-15-00030-s001.zip › clinpract-3436103-supplementary.pdf]

Supplementary Material

Addressing Rotator Cuff-Related Shoulder Pain: Findings from a Greek Regional Observational Study Utilizing a Clinical Case Scenario

*** Correspondence:** Corresponding Author: elparaskevop@uniwa.gr

Synthesis of best available evidence for clinical vignette (Retrieved from Smythe et al., 2021)

Clinical consideration	Recommended management for vignette	Rationale
Imaging	No imaging indicated	Imaging is not initially indicated in adult patients with limited movement and non-traumatic shoulder pain in the absence of red flags (Bussières et al., 2008). Imaging is only indicated in non-traumatic shoulder pain in the presence of red flag presentations, non-mechanical pain, and/or no response to an active treatment program after 4-6 weeks (Codsi and Howe, 2015; Diercks et al., 2014; Hopman et al., 2013 and Kulkarni et al., 2015).
Referral for surgical opinion	No referral indicated	Surgical referral is indicated if the patient experiences significant activity limitation and/or persistent pain and symptoms following engagement in a three month active, non-surgical treatment program (Codsi and Howe, 2015; Diercks et al., 2014; Hopman et al., 2013 and Kulkarni et al., 2015).
Referral for injection	No injection indicated	Heterogeneity on the timing and specific indication for corticosteroid injection for rotator cuff tendinopathy is evident in guidelines.<br>The only commonality between guidelines is that injection is recommended for pain relief, often only when pain is described as severe (Codsi and Howe, 2015; Diercks et al., 2014; Hopman et al., 2013 and Kulkarni et al., 2015).
Exercise treatment	An exercise program of at least a 12 week duration is indicated	Prescribed exercise programs are recommended as the initial treatment for rotator cuff tendinopathy (Codsi and Howe, 2015; Diercks et al., 2014; Hopman et al., 2013; Kulkarni et al., 2015 and Pedowitz et al., 2011). Exercise programs should be undertaken for a minimum of 12 weeks but may need to be continued for 6-12 months

		(Diercks et al., 2014; Hopman et al., 2013 and Kulkarni et al., 2015).
Education	Education is indicated	Education is recommended as an integral component of rotator cuff tendinopathy management (Codsi and Howe, 2015; Diercks et al., 2014; Hopman et al., 2013 and Kulkarni et al., 2015). Topics recommended include education on management, activity modification, beliefs and ideas that may impact patient outcome and education to address any relevant yellow flags (Codsi and Howe, 2015; Diercks et al., 2014; Hopman et al., 2013 and Kulkarni et al., 2015).
Adjunctive treatment	Manual therapy (massage and mobilisation) may be used in conjunction with an active treatment program<br><br>Paracetamol and/or NSAIDs are indicated as initial management	Manual therapy (massage and mobilisation) when combined with an active program may improve patient outcomes (Codsi and Howe, 2015; Diercks et al., 2014 and Hopman et al., 2013)<br><br>Electrotherapy modalities including therapeutic ultrasound, transcutaneous electromagnetic stimulation, low Level laser therapy and bipolar interferential current show no benefit for rotator cuff tendinopathy and are not indicated tendinopathy (Hopman et al., 2013 and Pedowitz et al., 2011).<br><br>Paracetamol should be prescribed as the initial choice for mild to moderate pain with the option of also being prescribed non-steroidal anti inflammatories (NSAIDs) (Diercks et al., 2014; Hopman et al., 2013; Kulkarni et al., 2015 and Pedowitz et al., 2011).
